# Supplementary material for: Hyodeoxycholic acid ameliorates nonalcoholic fatty liver disease by inhibiting RAN-mediated PPARα nucleus-cytoplasm shuttling
Source: Nat Commun. 2023 Sep 6;14:5451. doi: 10.1038/s41467-023-41061-8 (PMC10482907; doi:10.1038/s41467-023-41061-8)
Supplement: Supplementary file 1 — Supplementary Information [file 41467_2023_41061_MOESM1_ESM.pdf]

SUPPLEMENTARY INFORMATION FOR

Hyodeoxycholic acid ameliorates nonalcoholic fatty liver disease by inhibiting RAN-mediated PPARα nucleus-cytoplasm shuttling

Jing Zhong[1,2#], Xiaofang He[1#], Xinxin Gao[1], Qiaohong Liu[3], Yu Zhao[3], Ying Hong[1], Weize Zhu[1], Juan Yan[1], Yifan Li[1], Yan Li[1], Ningning Zheng[1], Yiyang Bao[1], Hao Wang[1], Junli Ma[1], Wenjin Huang[1], Zekun Liu[1], Yuanzhi Lyu[4], Xisong Ke[5], Wei Jia[6,7], Cen Xie[8*], Yiyang Hu[3*], Lili Sheng[1*], Houkai Li[1*]

Affiliations:

[1] School of Pharmacy, Shanghai University of Traditional Chinese Medicine, Shanghai, 201203, China.

[2]Huzhou Key Laboratory of Precision Medicine Research and Translation for Infectious Diseases, Affiliated Huzhou Hospital, Zhejiang University School of Medicine, Huzhou, 313000, China.

[3] Key Laboratory of Liver and Kidney Diseases (Ministry of Education), Institute of Liver Diseases, Shuguang Hospital Affiliated to Shanghai University of Traditional Chinese Medicine, Shanghai, 201203, China.

[4] Department of Dermatology, School of Medicine, University of California, Davis, Sacramento, CA, USA.

[5] Institute of Interdisciplinary Integrative Medicine Research, Shanghai University of Traditional Chinese Medicine, Shanghai, 201203, China.

[6] Center for Translational Medicine, Shanghai Sixth People's Hospital Affiliated to Shanghai Jiao Tong University School of Medicine, Shanghai, 200233, China.

[7] School of Chinese Medicine, Hong Kong Baptist University, Kowloon Tong, Hong Kong, 999077, China.

[8] State Key Laboratory of Drug Research, Shanghai Institute of Materia Medica, Chinese Academy of Sciences, Shanghai, 201203, China.

This PDF file includes:

Supplementary Figures 1 to 15
Supplementary Tables 1 to 2
Supplementary Methods

Supplementary Figure 1

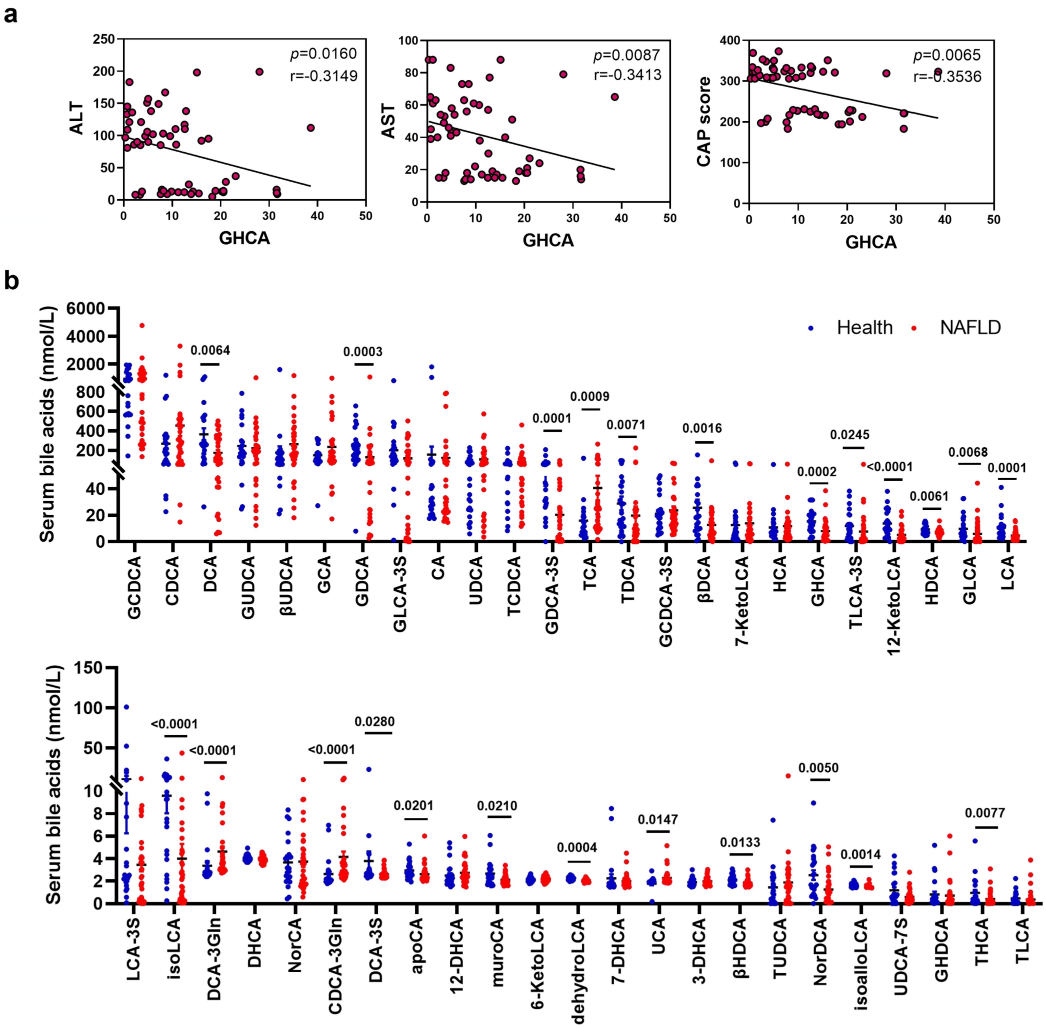

Figure S1. Bile acid profile in the serum of patients with NAFLD. a Scatter plots of serum GHCA with serum ALT, AST, and CAP score. **b** Concentration of BAs in the serum of health (*n*=24) and NAFLD (*n*=34) individuals. Data are presented as mean values ± SEM. Difference between groups were determined by unpaired two-tailed Mann-Whitney U test. Source data are provided as a Source Data file.

Supplementary Figure 2

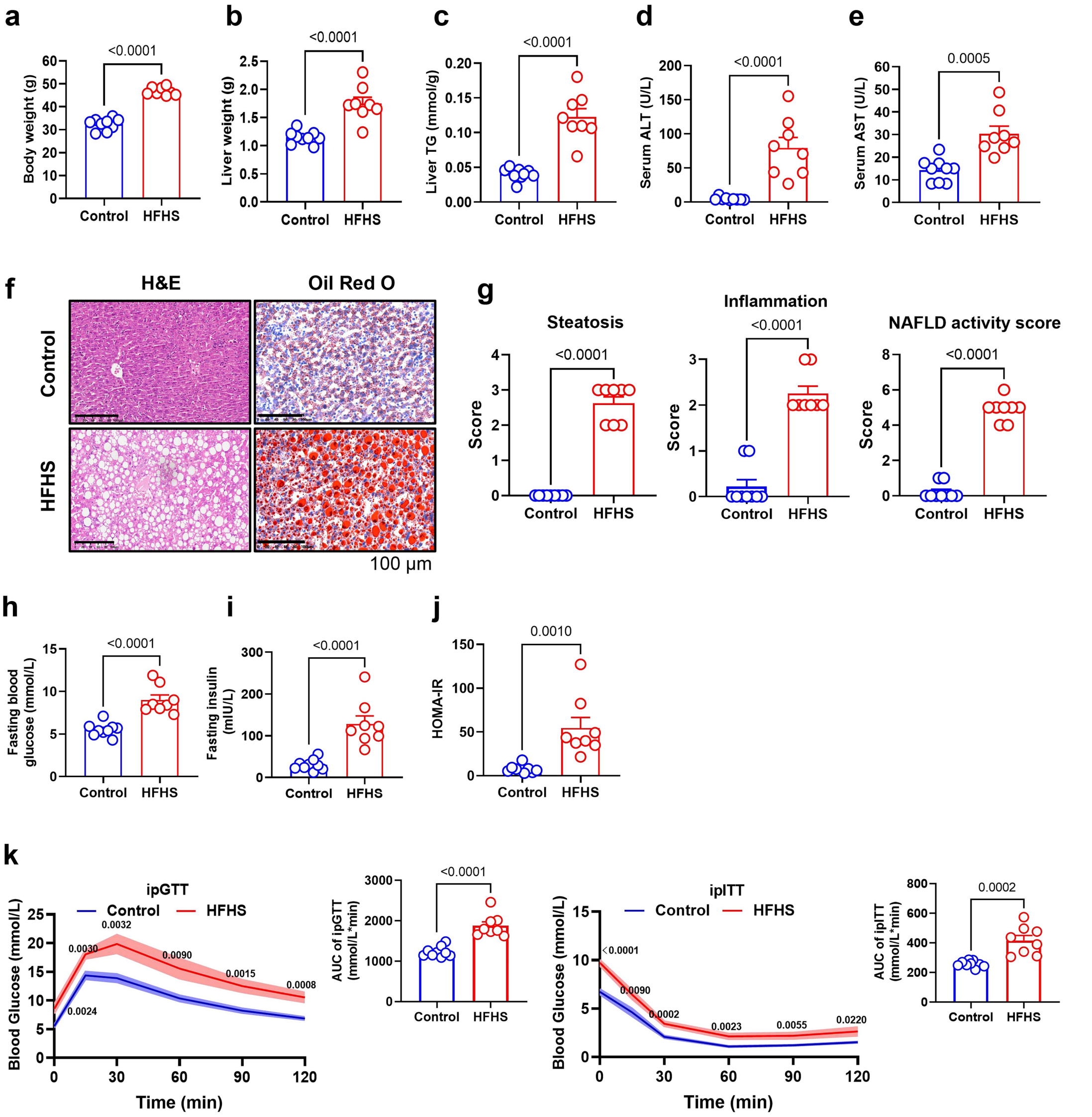

Figure S2. HFHS diet intake causes metabolic disorder in mice. C57BL/6 mice were fed a normal chow or HFHS diet for 24 weeks (*n*=9 for control, *n*=8 for HFHS). **a-e** Body weight, liver weight, liver TG, serum ATL, serum AST. **f** Representative images of liver H&E and Oil Red O staining (Scale bar, 100 μm). **g** Steatosis score, inflammation score, and NAFLD activity score. **h** Fasting blood glucose level. **i** Fasting insulin level. **j** HOMA-IR index. **k** ipGTT and ipITT results with area under curve (AUC) calculation.. Data are presented as mean values ± SEM. Difference between groups were determined by unpaired two-tailed Student's *t*-test. Source data are provided as a Source Data file.

Supplementary Figure 3

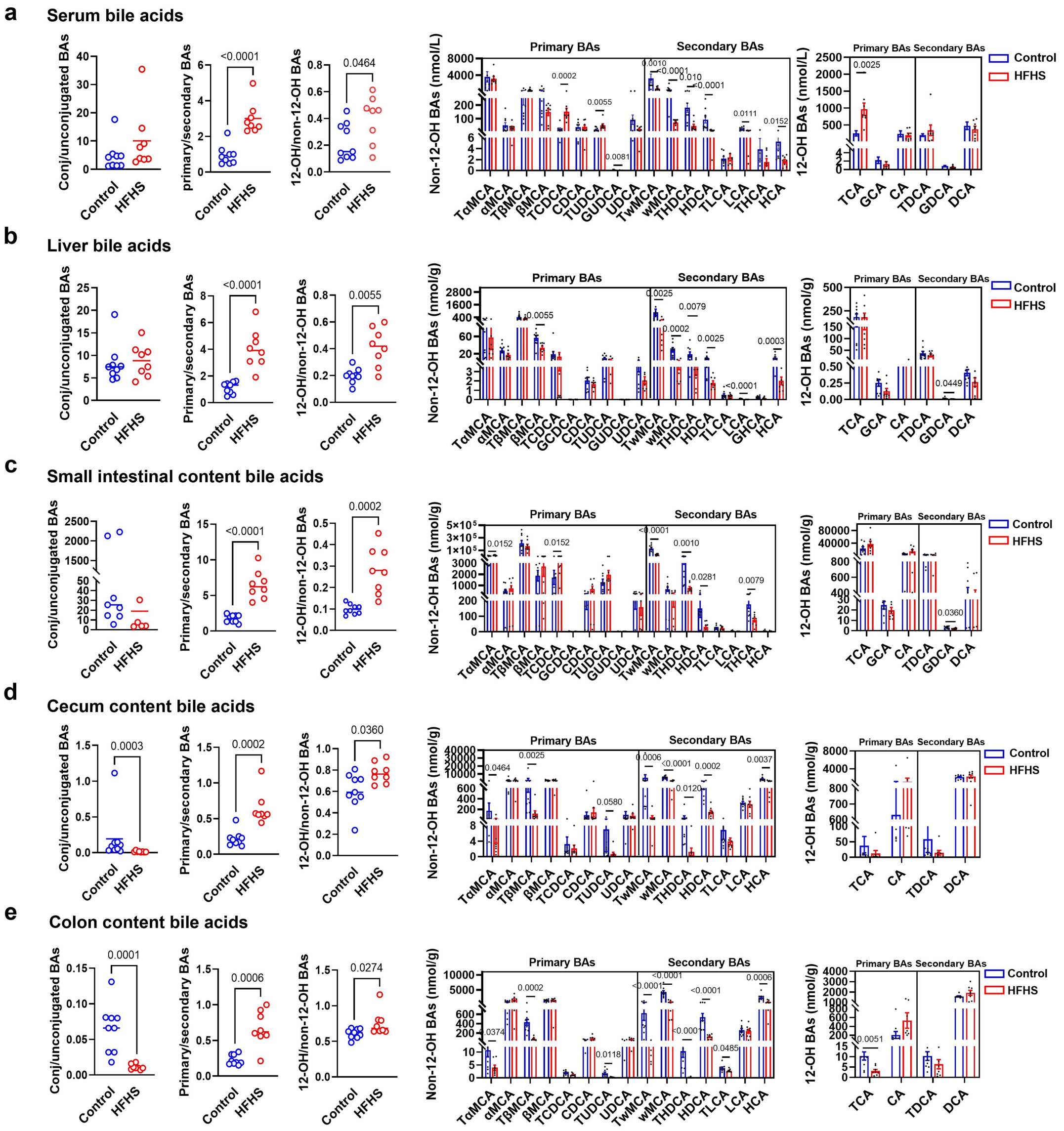

Figure S3. Bile acid profiles of HFHS-fed mice. Conjugated to unconjugated BAs ratio, primary to secondary BAs ratio, 12-OH to non-12-OH BAs ratio, and individual BAs level in the serum (**a**), liver (**b**), small intestinal content (**c**), cecum content (**d**), and colon content (**e**). (**a-e**), *n*=9 for control, *n*=8 for HFHS. Data are presented as mean values ± SEM. Difference between groups were determined by unpaired two-tailed Mann-Whitney U test. Source data are provided as a Source Data file.

Supplementary Figure 4

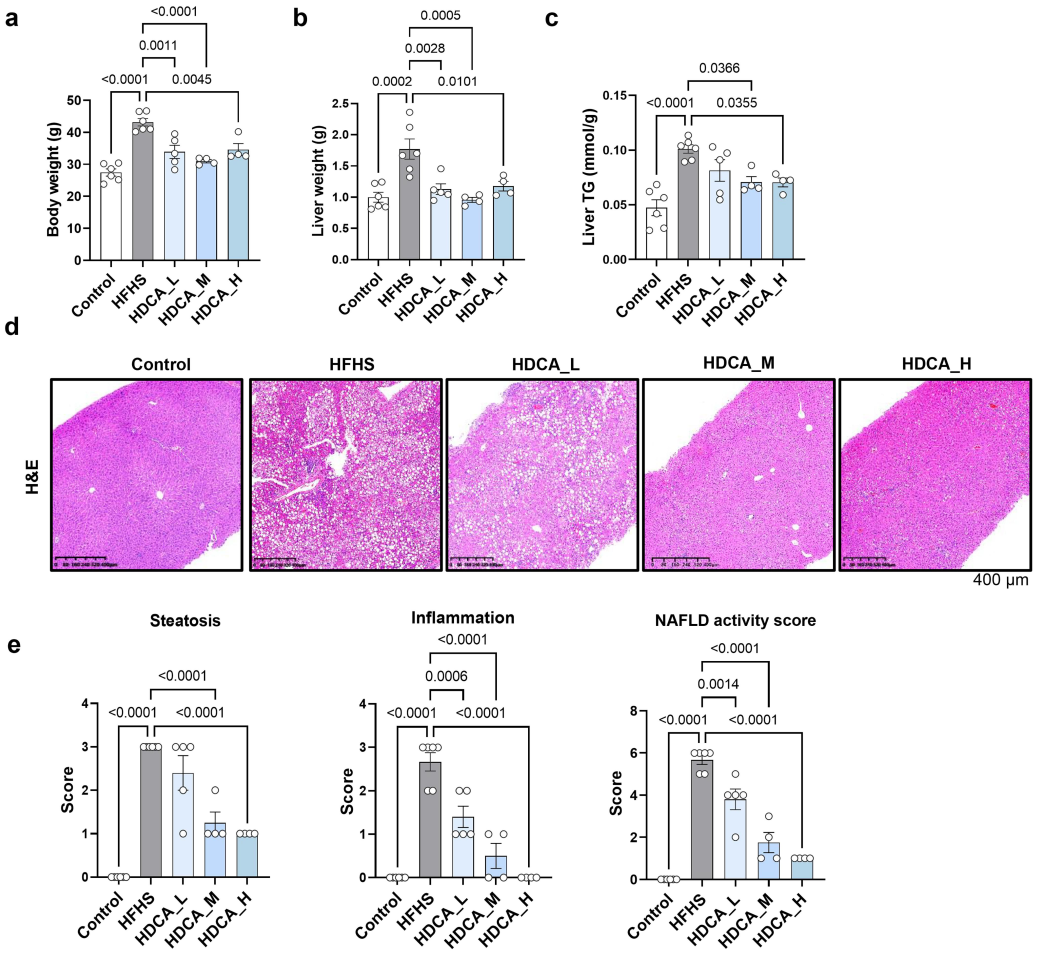

Figure S4. Dose-dependent effect of HDCA on ameliorating NAFLD in mice. C57BL/6 mice were fed a normal chow diet or HFHS diet for 4 weeks followed by another 8 weeks for HDCA intervention groups under HFHS feeding (HDCA-L, 0.3125% HDCA in HFHS diet; HDCA-M, 0.625% HDCA in HFHS diet; HDCA-H, 1.25% HDCA in HFHS diet). **a** Body weight. **b** Liver weight. **c** Liver TG level. **d** Representative images of liver H&E staining (Scale bar, 400 μm). **e** Steatosis score, inflammation score, and NAFLD activity score. (**a-e**), *n*=6 for Control and HFHS, *n*=5 for HDCA_L, *n*=4 for HDCA_M and HDCA_H. Data are presented as mean values ± SEM. Difference between groups were determined using one-way ANOVA test followed by Tukey's multiple comparison. Source data are provided as a Source Data file.

Supplementary Figure 5

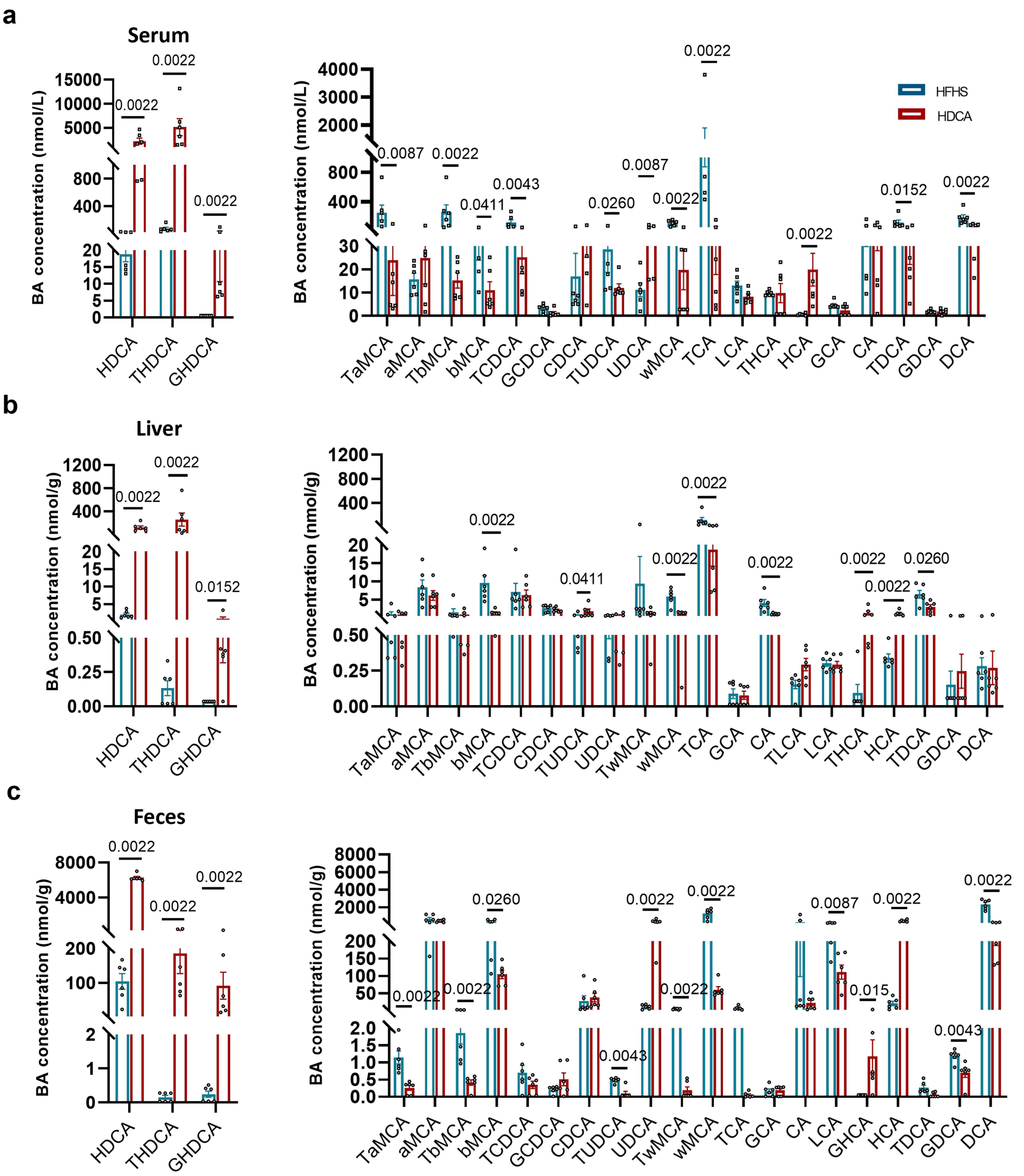

Figure S5. Bile acid profiles of HDCA-supplemented mice. Serum (**a**), liver (**b**), feces (**c**). (**a-c**), n=6 per group. Data are presented as mean values ± SEM. Difference between groups were compared using unpaired two-tailed Mann-Whitney U test. Source data are provided as a Source Data file.

Supplementary Figure 6

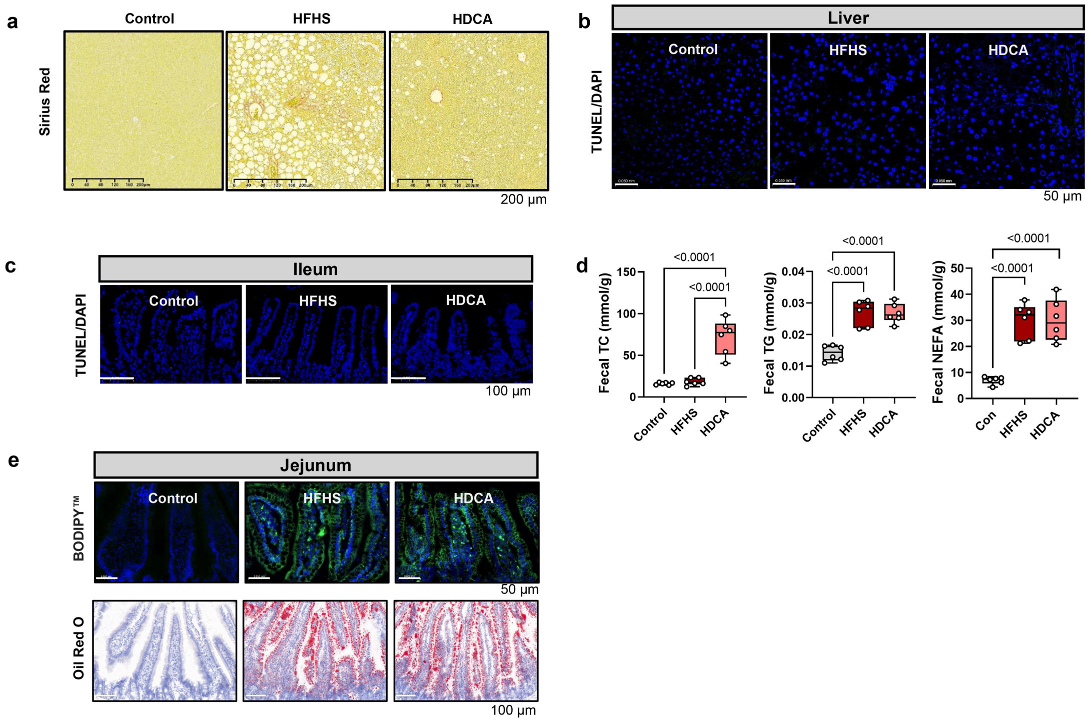

Figure S6. HDCA affects cholesterol absorption but not apoptosis. The C57BL/6 mice in control group and HFHS group were fed with chow or HFHS diet for 12 weeks. HDCA group were fed with HFHS for 4 weeks and then supplemented with 0.625% HDCA in the diet for another 8 weeks. **a** Representative images of Sirius red staining in liver (Scale bar, 200 μm) (*n*=6 mice/group). **b** Fluorescence images of TUNEL staining in the liver (Scale bar, 50 μm) (*n*=3 mice/group). **c** Fluorescence images of TUNEL staining in the ileum (Scale bar, 100 μm) (*n*=3 mice/group). **d** Level of fecal TC, TG, and NEFA (*n*=6 mice/group). **e** Fluorescence images of BODIPY (green) lipid absorbance in jejunum 2 h post-gavage. DAPI counterstains nucleic in blue (Scale bar, 50 μm) and Oil Red O staining of jejunum (Scale bar, 100 μm). The findings in (e) were confirmed in two independent experiments. Data are presented as mean values ± SEM. Difference between groups determined by one-way ANOVA test followed by Tukey's multiple comparison. Source data are provided as a Source Data file.

Supplementary Figure 7

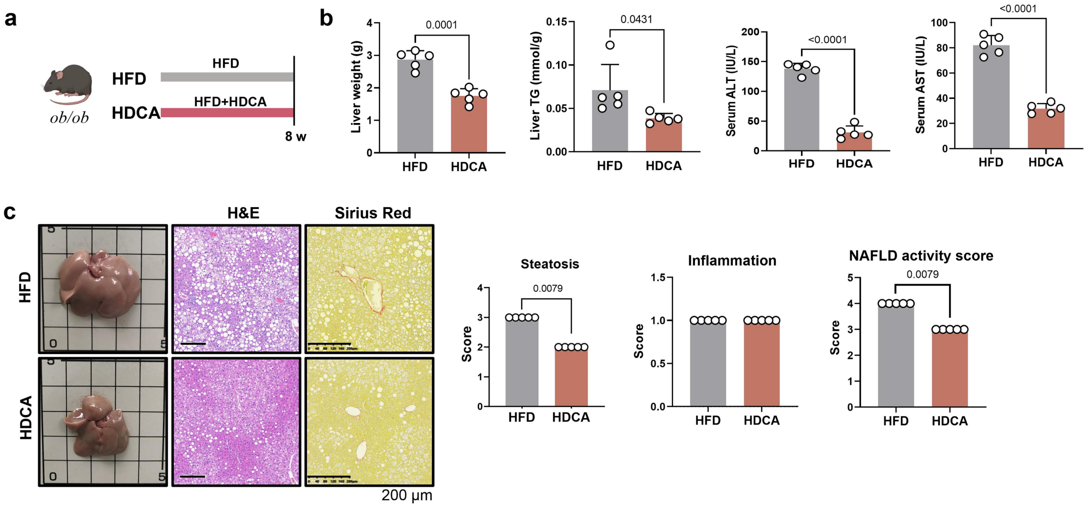

Figure S7. HDCA alleviates NAFLD in *ob/ob* mice. a Schematic of HDCA intervention in HFD-fed *ob/ob* mice. *Ob/ob* mice were fed a HFD diet (*n*=5) or HFD diet plus 1.25% HDCA in diet (*n*=5) for 8 weeks. Mouse element created with BioRender.com. **b** Liver weight, liver TG, serum ALT and serum AST. **c** Representative images of liver general appearance, H&E staining (Scale bar, 200 μm) and Sirius red staining (Scale bar, 200 μm), as well as steatosis score, inflammation score, and NAFLD activity score. Data are presented as mean values ± SEM. Difference between groups determined by unpaired two-tailed Student's *t*-test. Source data are provided as a Source Data file.

Supplementary Figure 8

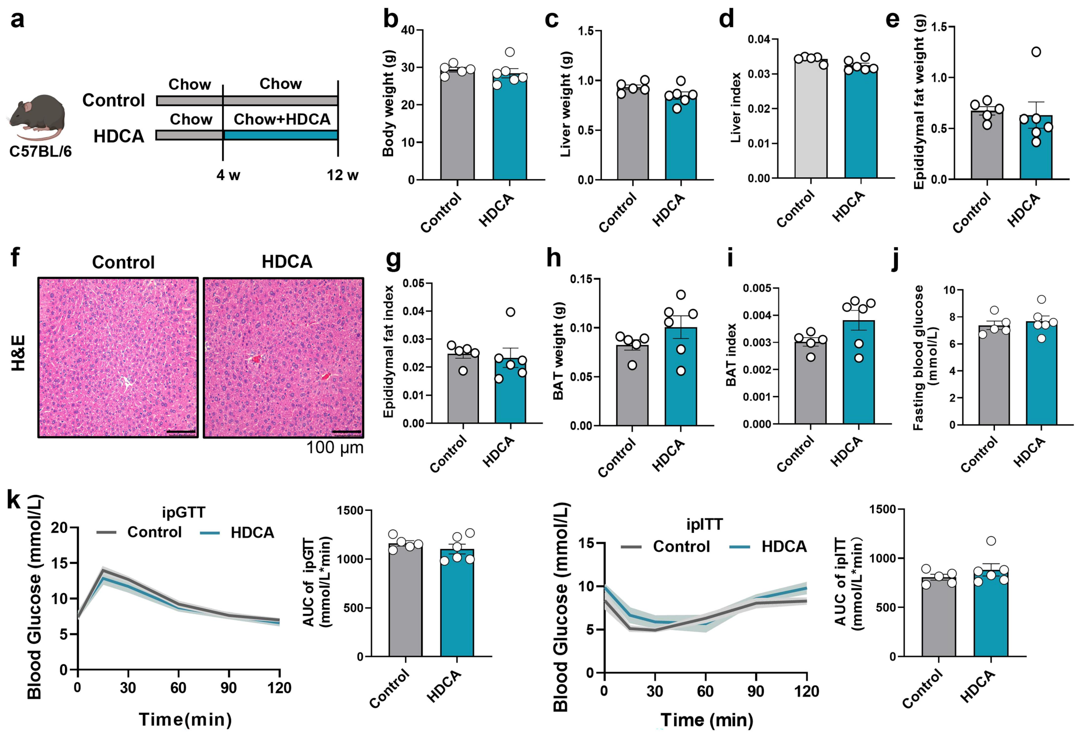

Figure S8. The effect of HDCA on chow diet-fed mice. a Schematic of HDCA intervention in chow diet-fed C57BL/6 mice. Mice were fed a normal chow diet for 4 weeks followed by with or without 0.625% HDCA supplementation for another 8 weeks (*n*=5 for Control, *n*=6 for HDCA). Mouse element created with BioRender.com. **b** Body weight. **c** Liver weight. **d** Liver index. **e** Epididymal fat weight. **f** Representative images of liver H&E staining (Scale bar, 100 μm). **g** Epididymal fat index. **h** BAT weight. **i** BAT index. **j** Fasting blood glucose level. **k** ipGTT and ipITT results with area under curve (AUC) calculation. Data are presented as mean values ± SEM. Difference between groups were compared by unpaired two-tailed Student's *t*-test. Source data are provided as a Source Data file.

Supplementary Figure 9

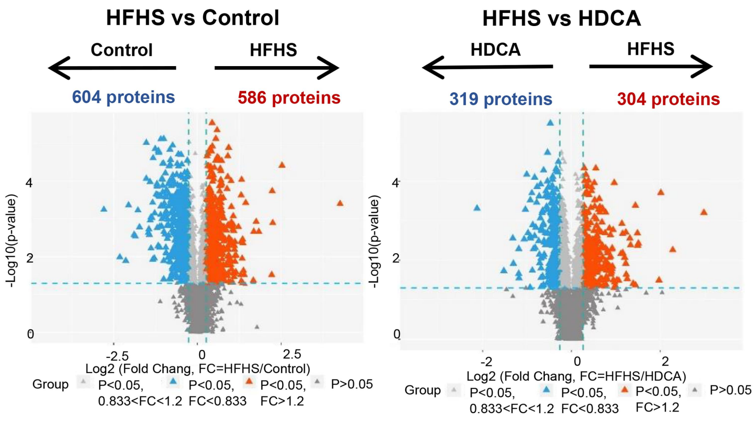

Figure S9. Volcano plot of hepatic proteomics results.

Supplementary Figure 10

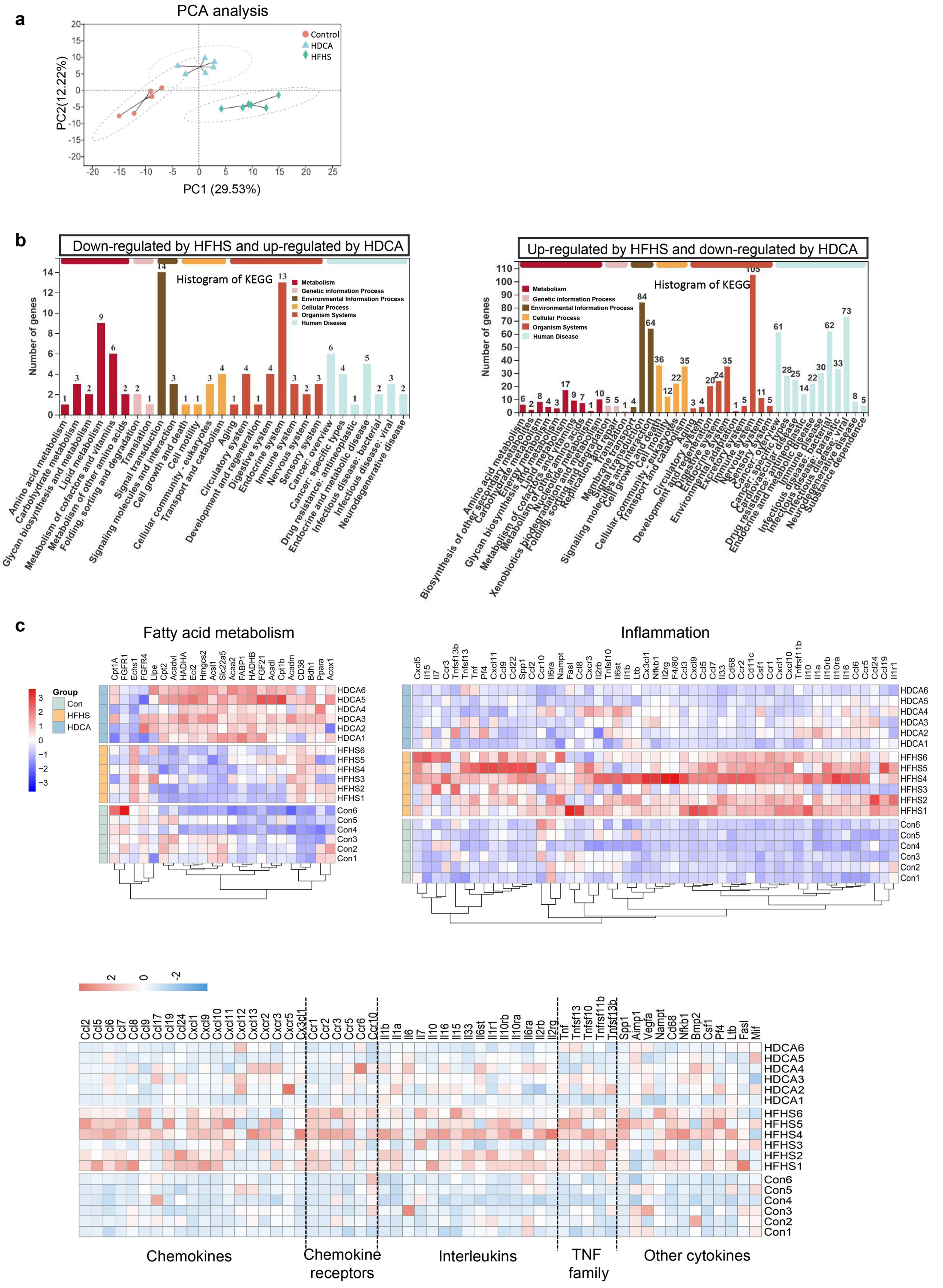

Fig. S10 Hepatic transcriptomics results. a PCA plots of liver gene profiles. **b** Histogram of KEGG functional annotation analysis of the reserved genes by HDCA intervention. **c** Heatmap for fatty acid metabolism and inflammation related genes.

Supplementary Figure 11

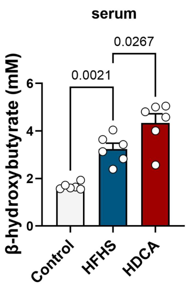

Figure S11. HDCA promotes liver ketogenesis. Mice are from Fig. 2a (*n*=6 per group). Data are presented as mean values ± SEM. Difference between groups were determined by one-way ANOVA test followed by Tukey's multiple comparison. Source data are provided as a Source Data file.

Supplementary Figure 12

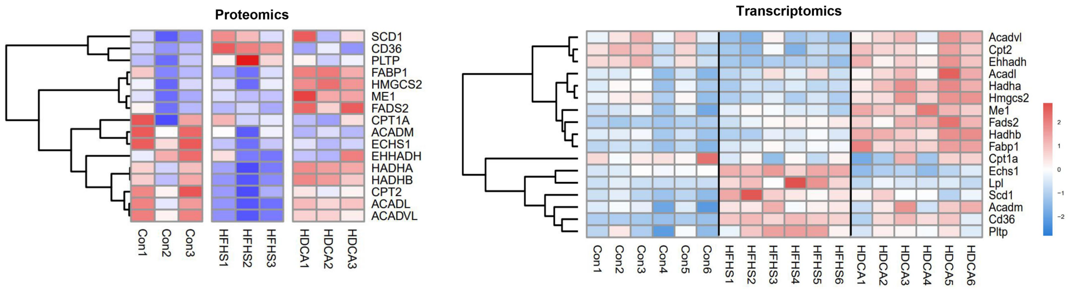

Fig. S12 The expression changes of PPARα target proteins and genes with HDCA treated mice as shown in figure 2a.

Supplementary Figure 13

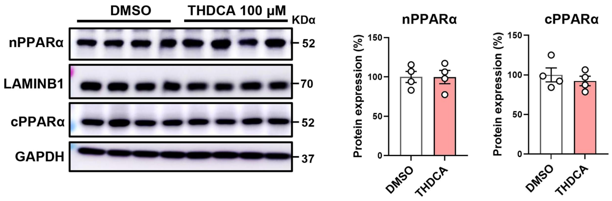

Figure S13. THDCA intervention can not promote the nuclear accumulation of PPARα. AML12 cells were treated with or without 100 μM THDCA for 24 h (*n*=4 per group). The findings were confirmed in three independent experiments. Data are presented as mean values ± SEM. Differences were determined by unpaired two-tailed Student's *t*-test. The average of protein expression in DMSO group is normalized as 100%. Source data are provided as a Source Data file.

Supplementary Figure 14

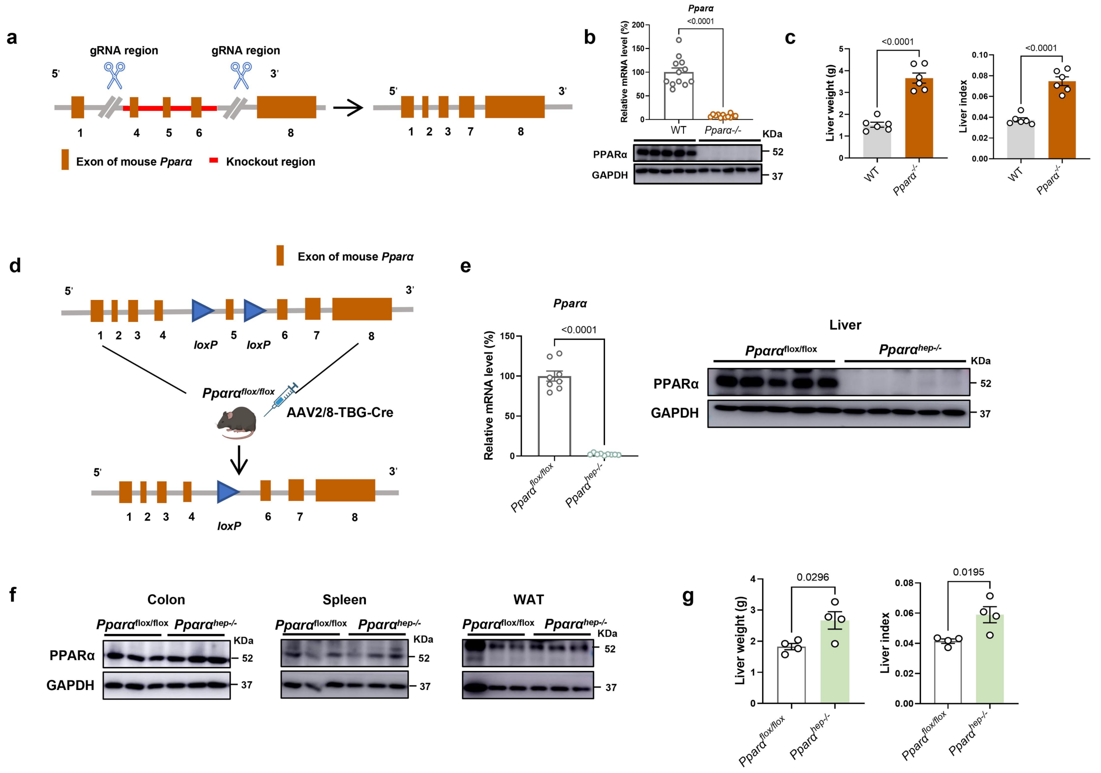

Figure S14. The identification of *Ppara*$^{-/-}$ and *Ppara*$^{hep-/-}$ mice. a Schematic representation of whole body *Ppara* gene deletion. **b** Confirmation of the *Ppara*$^{-/-}$ mice at mRNA (*n*=12 per group) and protein levels (*n*=5 per group). **c** Liver weight and liver index of WT and *Ppara*$^{-/-}$ mice under HFHS-fed condition (*n*=6 per group). **d** Schematic representation of *Ppara* gene deletion in liver. Mouse and syringe elements created with BioRender.com. **e** Confirmation of the *Ppara*$^{hep-/-}$ mice at mRNA (*n*=8 for *Ppara*$^{flox/flox}$, *n*=9 for *Ppara*$^{hep-/-}$) and protein levels (*n*=5 per group) in liver. **f** Confirmation of the *Ppara*$^{hep-/-}$ mice at protein levels in tissue of colon, spleen, WAT (*n*=3 per group). **g** Liver weight and liver index of *Ppara*$^{flox/flox}$ and *Ppara*$^{hep-/-}$ mice under HFHS-fed condition (*n*=4 per group). Data are presented as mean values ± SEM. Difference between groups were determined by unpaired two-tailed Student's *t*-test. The average of gene expression in WT or *Ppara*$^{flox/flox}$ group is normalized as 100%. Source data are provided as a Source Data file.

Supplementary Figure 15

```
Mouse-RAN   MAAQGEPQVQFKLVLVGDGGTGKTTFVKRHLTGEFEKKYVATLGVEVHPLVFHTNRGPIKFNVWDTAGQEKFGGLRDGYY  80
Human-RAN1  MAAQGEPQVQFKLVLVGDGGTGKTTFVKRHLTGEFEKKYVATLGVEVHPLVFHTNRGPIKFNVWDTAGQEKFGGLRDGYY  80
Human-RAN2  MTAQGEPQVQFKLVLVGDGGTGKTTFVKRHLTGEFEKKYVATLGVEVHPLVFHTNRGPIKFNVWDTAGQEKFGGLRDGYY  80

Mouse-RAN   IQAQCAIIMFDVTSRVTYKNVPNWHRDLVRVCENIPIVLCGNKVDIKDRKVKAKSIVFHRKKNLQYYDISAKSNYNFEKP  160
Human-RAN1  IQAQCAIIMFDVTSRVTYKNVPNWHRDLVRVCENIPIVLCGNKVDIKDRKVKAKSIVFHRKKNLQYYDISAKSNYNFEKP  160
Human-RAN2  IQAQCAIIMFDVTSRVTYKNVPNWHRDLVRVCENIPIVLCGNKVDIKDRKVKAKSIVFHRKKNLQYYDISAKSNYNFEKP  160

Mouse-RAN   FLWLARKLIGDPNLEFVAMPALAPPEVVMDPALAAQYEHDLEVAQTTALPDEDDDL  216
Human-RAN1  FLWLARKLIGDPNLEFVAMPALAPPEVVMDPALAAQYEHDLEVAQTTALPDEDDDL  216
Human-RAN2  FLWLARKLIGDPNLEFVAMPALAPPEVVMDPALAAQYEHDLEVAQTTALPDEDDDL  216
```

Fig. S15 Blast of the amino acid sequence of RAN protein in mouse and human. Human-RAN1 and Human-RAN2 referred to JHU01210B3C3 and JHU15167B10C25 in the HuProt^{TM} 20K Human Proteome Microarrays, respectively.

Supplementary Table 1. The characteristics of NAFLD and healthy subjects

**Index**	**HC (n=24)**	**NAFLD (n=34)**	**p.value**
Age (year)	27.6±2.4	38.9±8.6	<0.0001
Female	1(4.2)	4(11.8)	0.3851
BMI	20.7±1.8	30.0±3.2	<0.0001
TC (nmol/L)	4.5±0.7	5.3±0.9	0.0031
TG (nmol/L)	0.7±0.3	2.3±1.5	<0.0001
HDL-C (nmol/L)	1.5±0.3	1.1±0.2	<0.0001
LDL-C (nmol/L)	2.4±0.5	3.2±0.7	<0.0001
ALT (U/L)	13.7±7.0	121.3±32.8	<0.0001
AST (U/L)	17.6±3.7	59.1±15.6	<0.0001
ALB (U/L)	44.5±2.1	47.3±2.5	<0.0001
FBG (mmol/L)	4.5±0.5	4.9±0.5	0.0009
CAP score	212.3±15.3	326.3±17.7	<0.0001
Antilipemic drugs	0	1 (2.9)	>0.9999
Hepatoprotectants	0	3 (8.8)	0.2556
Antihypertensive drugs	0	4 (11.8)	0.1344

Note: Difference of age, BMI, TC, TG, HDL-C, LDL-C, ALT, AST, ALB, FBG, Fireoscan score bwtween groups were determined by two-tailed Student's *t* test. Difference of sex, drug taking between groups were determined by two-sided Fisher's exact test.

Supplementary Table 2.The primers for qPCR

Gene	Forward sequence (5' to 3')	Reverse sequence (5' to 3')
Pparα KO	ACCACTACGGAGTTCACGCATG	GAATCTTGCAGCTCCGATCACAC
Pparα CKO	CCCTGAACATCGAGTGTCGAA	TTCGCCGAAAGAAGCCCTTA
Ccl2	CTTCTGGGCCTGCTGTTCA	CCAGCCTACTCATTGGGATCA
Ccl9	TCCAGAGCAGTCTGAAGGCACA	CCGTGAGTTATAGGACAGGCAG
Cxcl1	TCCAGAGCTTGAAGGTGTTGCC	AACCAAGGGAGCTTCAGGGTCA
Cxcl9	CCTAGTGATAAGGAATGCACGATG	CTAGGCAGGTTTGATCTCCGTTC
Cxcl10	GAAATCATCCCTGCGAGCCTATC	GCTAAACGCTTTCATTAAATTCTTG
Cxcl11	CCGAGTAACGGCTGCGACAAAG	CCTGCATTATGAGGCGAGCTTG
TNF-α	TGATCCGAGATGTGGAACTG	CACGAGCAGGAATGAGAAGA
IL-6	CTCTCCGCAAGAGACTTCCA	CCTCCGACTTGTGAAGTGGT
IL-1β	CACAGCAGCATCTCGACAAG	CCTGCAGTGCAGCTGTCTAA
Col3a1	ACGTAAGCACTGGTGGACAG	CCGGCTGGAAAGAAGTCTGA
Shp	GGAGTCTTTCTGGAGCCTTG	ATCTGGGTTGAAGAGGATCG
Cpt2	CAGCACAGCATCGTACCCA	TCCCAATGCCGTTCTCAAAAT
Fabp1	TAGGTCTGCCCGAGGAC	CCAGGGTGAACTCATTGC
Hadhb	TGAATATGCACTGCGTTCTCAT	CCTTTCCTGGTACTTTGAAGGG
Col1a1	TGCTGGTCCTGCTGGTC	CCTTGTTCGCCTGTCTCAC
Cpt1	TGAGTGGCGTCCTCTTTGG	CAGCGAGTAGCGCATAGTCATG
Acacl	TGCCCTATATTGCGAATTACGG	CTATGGCACCGATACACTTGC
Acadm	AGGGTTTAGTTTTGAGTTGACGG	CCCCGCTTTTGTCATATTCCG
Acadvl	TGACCTTGGTGTTAGCGTTAC	CTGGGCCTTTGTGCCATAGAG
18S	CCATCCAATCGGTAGTAGCG	GTAACCCGTTGAACCCCATT

Supplementary Methods

The recruitment of NAFLD and healthy subjects

Samples of NAFLD subjects and clinical data were from a multicenter, randomized, double-blind clinical trial conducted at four centers in Shanghai, China, in patients with imaging confirmed NAFLD with abnormal liver function. The aim of this trial was to evaluate the efficacy of a traditional Chinese medicine prescription in patients with NAFLD. The samples/data of NAFLD patients were before the intervention of the traditional Chinese medicine. This trial was approved by the Institutional Review Board of Shuguang Hospital Affiliated to Shanghai University of Chinese Medicine (Approval No. 2017-548-31) and was conducted in accordance with the Principles of Good Clinical Practice and the Declaration of Helsinki. Subjects were recruited mainly from outpatient clinics. Recruitment advertisements reviewed by the Ethics Committee were placed in places where potential subjects congregate to facilitate recruitment. All clinical examinations in this clinical trial were free of charge, and written informed consent is obtained from each patient prior to screening and enrollment.

Healthy subjects were recruited from the Phase I clinical program of Good Clinical Practice Center or the Physical Examination Center of Shanghai Shuguang Hospital. This study was conducted in accordance with the Declaration of Helsinki and was approved by Institutional Review Board of Shuguang Hospital Affiliated to Shanghai University of Chinese Medicine (No. 2019-662-17-01).

The inclusion and exclusion criteria for NAFLD patients and healthy subjects

The diagnostic criteria for NAFLD were referred to the Guidelines for the Diagnosis and Treatment of Non-alcoholic Fatty Liver Disease (2010) issued by Fatty liver and Alcoholic Liver Disease Group, Hepatology Society of Chinese Medical Association.

The inclusion criteria for NAFLD participants: (1) Meet the above diagnostic criteria for NAFLD; (2) 18-65 years old; (3) CAP score > 300 (ECHOSENS, FibroScan 502); (4) Serum alanine aminotransferase (ALT) >80.

Exclusion criteria for NAFLD participants: (1) Excessive alcohol consumption (140 g/week in men or 70 g/week in women); (2) Combined with alcoholic liver disease, viral hepatitis, Wilson's disease, autoimmune liver disease, or other chronic liver disease; (3) Combined with hypothyroidism, inflammatory bowel disease, Cushing's syndrome, lack of beta lipoprotein hematic disease, encephalopathy, type of lipid deposition disease, fatty liver tumor and some associated with insulin resistance syndrome (lipid atrophic diabetes, Mauriac syndrome); (4) Medicine with drugs that can cause fatty liver, such as tamoxifen, ethamiodarone, valproate, glucocorticoids, methotrexate, etc. or total parenteral nutrition; (5) Combined with other serious diseases include renal insufficiency, heart disease, lung disease, malignant tumors of the liver and other systems, mental illness, and other conditions affecting the metabolic state of the whole body, such as pregnancy, breastfeeding, etc; (6) Antibiotics and proton pump inhibitors were used within a month; (7) Medicine with drugs for lowering triglycerides or cholesterol within three months, such as kinds of statin or fibrate;(8) Gastrointestinal surgery was performed in the last year or weight-loss medications taken with more than 10 percent of body weight lost.

Healthy controls were defined as individuals with normal routine laboratory tests and no diagnosis of metabolic diseases such as hypertension, diabetes, hyperlipidemia, hyperuricemia, and other serious conditions.
