# Supplementary material for: Hyodeoxycholic acid ameliorates nonalcoholic fatty liver disease by inhibiting RAN-mediated PPARα nucleus-cytoplasm shuttling
Source: Nat Commun. 2023 Sep 6;14:5451. doi: 10.1038/s41467-023-41061-8 (PMC10482907; doi:10.1038/s41467-023-41061-8)
Supplement: Supplementary file 3 — Reporting Summary [file 41467_2023_41061_MOESM3_ESM.pdf]

nature portfolio

Corresponding author(s): Houkai Li
Last updated by author(s): Aug 18, 2023

# Reporting Summary

Nature Portfolio wishes to improve the reproducibility of the work that we publish. This form provides structure for consistency and transparency in reporting. For further information on Nature Portfolio policies, see our Editorial Policies and the Editorial Policy Checklist.

## Statistics

For all statistical analyses, confirm that the following items are present in the figure legend, table legend, main text, or Methods section.

| n/a | Confirmed | |
|---|---|---|
| ☐ | ☒ | The exact sample size (*n*) for each experimental group/condition, given as a discrete number and unit of measurement |
| ☐ | ☒ | A statement on whether measurements were taken from distinct samples or whether the same sample was measured repeatedly |
| ☐ | ☒ | The statistical test(s) used AND whether they are one- or two-sided<br>*Only common tests should be described solely by name; describe more complex techniques in the Methods section.* |
| ☒ | ☐ | A description of all covariates tested |
| ☐ | ☒ | A description of any assumptions or corrections, such as tests of normality and adjustment for multiple comparisons |
| ☐ | ☒ | A full description of the statistical parameters including central tendency (e.g. means) or other basic estimates (e.g. regression coefficient) AND variation (e.g. standard deviation) or associated estimates of uncertainty (e.g. confidence intervals) |
| ☐ | ☒ | For null hypothesis testing, the test statistic (e.g. *F*, *t*, *r*) with confidence intervals, effect sizes, degrees of freedom and *P* value noted<br>*Give P values as exact values whenever suitable.* |
| ☒ | ☐ | For Bayesian analysis, information on the choice of priors and Markov chain Monte Carlo settings |
| ☒ | ☐ | For hierarchical and complex designs, identification of the appropriate level for tests and full reporting of outcomes |
| ☒ | ☐ | Estimates of effect sizes (e.g. Cohen's *d*, Pearson's *r*), indicating how they were calculated |

*Our web collection on statistics for biologists contains articles on many of the points above.*

## Software and code

Policy information about availability of computer code

| | |
|---|---|
| Data collection | The histology images were acquired by Leica Aperio or ImageScope Digital slice scanner (3D Histech, Pannoramic MIDI, Hungary). Multifunctional microplate detector (TECAN SPARK) and the Amersham Imager 600 (GE, USA) were used for Chemiluminescence imaging. QuantStudio5 (Applied Biosystem) was used for RT-PCR. Digital slice scanner (3D Histech, Pannoramic MIDI, Hungary) was used for fluorescence staining in tissues. Leica TCS SP8 Confocal Microscopy was used in PPARα immunofluorescence in nucleus, and in PLA assay. SpectraMax M5e (Molecular Devices, USA) was used for luciferase reporter gene assays. The high-throughput RNA sequencing was performed via Illumina HiSeq XTEN /NovaSeq 6000 squencer. The proteome analysis was performed on an Orbitrap Q-Exactive (Thermo Fisher Scientific) platform connected to an online nanoflow EASY nLC1200 HPLC system (Thermo Fisher Scientific). GenePixTM 4000B (Axon Instruments, CA) was used for human proteome microassay scanning.Roche ACCU-CHEK Performa was applied for blood glucose data collection. |
| Data analysis | The softwares of MassLynx v4.1 and TargetLynx V4.1 (Waters Corp., Milford, USA) were used for bile acid data process. SeqPrep, Sickle, HISAT2 and DESeq2 software were used for transcriptomics. Raw mass spectrometry data of proteomics were processed using Maxquant 1.6.5.0 (Thermo Scientific, USA). Docking analysis was carried out by Swiss-Dock software from the Swiss Institute of Bioinformatics (http://www.swissdock.ch/). GenePixTM Pro v6.0 was used for human proteome microassay data analysis. ImageJ(v1.8.0) was responsible for semi-quantitative immunofluorescence analysis of PPARα expression. R (v4.2.2), Graphpad Prism (v9), SPSS (v21.0) were used for statistical analysis. |

For manuscripts utilizing custom algorithms or software that are central to the research but not yet described in published literature, software must be made available to editors and reviewers. We strongly encourage code deposition in a community repository (e.g. GitHub). See the Nature Portfolio guidelines for submitting code & software for further information.

## Data

Policy information about availability of data

All manuscripts must include a data availability statement. This statement should provide the following information, where applicable:

- Accession codes, unique identifiers, or web links for publicly available datasets
- A description of any restrictions on data availability
- For clinical datasets or third party data, please ensure that the statement adheres to our policy

A data availability statement is included in the manuscript. The mass spectrometry proteomics data have been deposited to the ProteomeXchange Consortium via the iProX partner repository with the dataset identifier PXD044613. RNA sequencing data have been deposited in Genome Sequence Archive (GSA) under the accession code CRA012267. Human proteome microarray data have been deposited in GEO database under the accession code GSE241065. The protein structure of RAN was obtained from human Uniprot database (UniProt: P62827). Source data are provided with this paper.

## Human research participants

Policy information about studies involving human research participants and Sex and Gender in Research.

| | |
|---|---|
| Reporting on sex and gender | 24 healthy subjects (23 males and 1 female) and 34 NAFLD subjects (30 males and 4 females) were included in this study. Sex was not considered in study design, because the purpose of this study is to compare bile acid profile between NAFLD patients and healthy controls. |
| Population characteristics | A group of 58 subjects including 24 healthy subjects (23 males and 1 female, age:27.6±2.4 years old) and 34 NAFLD subjects (30males and 4 females, age: 38.9±8.6 years old) were included in this study. |
| Recruitment | Written informed consent was obtained from all participants before recruiment.<br>Samples of NAFLD subjects and clinical data were from a multicenter, randomized, double-blind clinical trial conducted at four centers in Shanghai, China, in patients with imaging confirmed NAFLD with abnormal liver function. The aim of this trial was to evaluate the efficacy of a traditional Chinese medicine prescription in patients with NAFLD. The samples/data of NAFLD patients were before the intervention of the traditional Chinese medicine. This trial was approved by the Institutional Review Board of Shuguang Hospital Affiliated to Shanghai University of Chinese Medicine (Approval No. 2017-548-31) and was conducted in accordance with the Principles of Good Clinical Practice and the Declaration of Helsinki. Subjects were recruited mainly from outpatient clinics. Recruitment advertisements reviewed by the Ethics Committee are placed in places where potential subjects congregate to facilitate recruitment. All clinical examinations in this clinical trial were free of charge with no participant compensation, and written informed consent is obtained from each patient prior to screening and enrollment.<br>Healthy subjects were recruited from the Phase I clinical program of GCP Center or the Physical Examination Center of Shanghai Shuguang Hospital. This study was conducted in accordance with the Declaration of Helsinki and was approved by Institutional Review Board of Shuguang Hospital Affiliated to Shanghai University of Chinese Medicine (No. 2019-662-17-01).<br><br>The diagnostic criteria for NAFLD were referred to the Guidelines for the Diagnosis and Treatment of Non-alcoholic Fatty Liver Disease (2010) issued by Fatty liver and Alcoholic Liver Disease Group, Hepatology Society of Chinese Medical Association.The inclusion criteria for NAFLD participants: (1) Meet the above diagnostic criteria for NAFLD; (2) 18-65 years old; (3) CAP score > 300 (ECHOSENS, FibroScan 502); (4) Serum alanine aminotransferase (ALT) >80. Exclusion criteria for NAFLD participants: (1) Excessive alcohol consumption (140 g/week in men or 70 g/week in women); (2) Combined with alcoholic liver disease, viral hepatitis, Wilson's disease, autoimmune liver disease, or other chronic liver disease; (3) Combined with hypothyroidism, inflammatory bowel disease, Cushing's syndrome, lack of beta lipoprotein hematic disease, encephalopathy, type of lipid deposition disease, fatty liver tumor and some associated with insulin resistance syndrome (lipid atrophic diabetes, Mauriac syndrome); (4) Medicine with drugs that can cause fatty liver, such as tamoxifen, ethamiodarone, valproate, glucocorticoids, methotrexate, etc. or total parenteral nutrition; (5) Combined with other serious diseases include renal insufficiency, heart disease, lung disease, malignant tumors of the liver and other systems, mental illness, and other conditions affecting the metabolic state of the whole body, such as pregnancy, breastfeeding, etc; (6) Antibiotics and proton pump inhibitors were used within a month; (7) Medicine with drugs for lowering triglycerides or cholesterol within three months, such as kinds of statin or fibrate;(8) Gastrointestinal surgery was performed in the last year or weight-loss medications taken with more than 10 percent of body weight lost.<br>Healthy controls were defined as individuals with normal routine laboratory tests and no diagnosis of metabolic diseases such as hypertension, diabetes, hyperlipidemia, hyperuricemia, and other serious conditions. |
| Ethics oversight | These protocols were approved by Institutional Review Board of Shuguang Hospital Affiliated to Shanghai University of Chinese Medicine (Approval No. 2017-548-31, No. 2019-662-17-01) |

Note that full information on the approval of the study protocol must also be provided in the manuscript.

# Field-specific reporting

Please select the one below that is the best fit for your research. If you are not sure, read the appropriate sections before making your selection.

- [x] Life sciences
- [ ] Behavioural & social sciences
- [ ] Ecological, evolutionary & environmental sciences

For a reference copy of the document with all sections, see nature.com/documents/nr-reporting-summary-flat.pdf

# Life sciences study design

All studies must disclose on these points even when the disclosure is negative.

| | |
|---|---|
| Sample size | All clinical samples that met the inclusion and exclusion criteria were analyzed for this study. We used as many as we could obtain. For animal experiments, we generally set 6 mice per group, which is a very common number of animals used, consistent with previous published papers (PMID: 37443159, PMID: 37142604). In addition, the number of liver knockout animals in each group is 4-5 per group, because the time for revising the article is limited, we only have those number of mice at that time. |
| Data exclusions | No data were excluded from analysis. |
| Replication | Indicated in figure legends. |
| Randomization | The mice were randomly divided and allocated into experimental groups.Cells were grown under the same conditions and randomly allocated into different treatment groups without any bias. |
| Blinding | The investigators were blinded to the group allocation during data collection. |

# Reporting for specific materials, systems and methods

We require information from authors about some types of materials, experimental systems and methods used in many studies. Here, indicate whether each material, system or method listed is relevant to your study. If you are not sure if a list item applies to your research, read the appropriate section before selecting a response.

## Materials & experimental systems

| n/a | Involved in the study | |
|---|---|---|
| ☐ | ☒ | Antibodies |
| ☐ | ☒ | Eukaryotic cell lines |
| ☒ | ☐ | Palaeontology and archaeology |
| ☐ | ☒ | Animals and other organisms |
| ☒ | ☐ | Clinical data |
| ☒ | ☐ | Dual use research of concern |

## Methods

| n/a | Involved in the study | |
|---|---|---|
| ☒ | ☐ | ChIP-seq |
| ☒ | ☐ | Flow cytometry |
| ☒ | ☐ | MRI-based neuroimaging |

## Antibodies

Antibodies used

1. Anti-PPARα antibody Abcam ab126285 WB: 1:1000 IF: 1:100
2. Anti-PPARα antibody Abcam ab215270 WB: 1:1000 IF: 1:100
3. Anti- CD36 antibody Abcam ab133625 WB: 1:1000
4. FABP1 Polyclonal antibody Proteintech 13626-1-AP WB: 1:1000
5. CPT1A antibody Cell singaling Technology #12252S WB: 1:1000
6. CPT2 Polyclonal antibody Proteintech 26555-1-AP WB: 1:1000
7. HMGCS2 antibody Cell singaling Technology #20940S WB: 1:1000
8. NF-κB p65 antibody Cell singaling Technology #8242S WB: 1:1000
9. Phospho-NF-κB p65 antibody Cell singaling Technology #3033S WB: 1:1000
10. CRM1 antibody Santa Cruz Biotechnology sc-74454 WB: 1:500 IP: 1:25 PLA: 1:10
11. RAN antibody Santa Cruz Biotechnology sc-271376 WB: 1:500 PLA: 1:20
12. GAPDH Monoclonal antibody Proteintech 60004-1-Ig WB: 1:5000

13 Anti- Lamin B1 antibody Abcam ab229025 WB: 1:1000

14. β-Actin antibody Cell singaling Technology #4970 WB: 1:1000
15. Mouse monoclonal anti-Flag Sigma-Aldrich F1804 WB: 1:1000 IP: 1:50
16. HA tag Polyclonal antibody Proteintech 51064-2-AP WB: 1:1000 IP: 1:50
17. Rabbit Control IgG ABclonal AC005 IP: 1:100

18. Mouse Control IgG ABclonal AC011 IP: 1:100
19 HRP Goat Anti-Rabbit IgG (H+L) ABclonal AS014 WB: 1:5000
20. Anti-mouse IgG, HRP-linked Antibody Cell singaling 7076 WB: 1:5000
21. ABflo® 594-conjugated Goat Anti-Rabbit IgG (H+L) ABclonal AS039 IF: 1:100
22. ABflo® 488-conjugated Goat Anti-Rabbit IgG (H+L) ABclonal AS053 IF: 1:100
23. CRM1/XPO1 Rabbit mAb ABclonal A19625 PLA: 1:10
24. PPARA Polyclonal antibody Proteintech 15540-1-AP PLA: 1:25

| | |
|---|---|
| Validation | 1. Anti-PPARα antibody https://www.abcam.cn/products/primary-antibodies/ppar-alpha-antibody-ab126285.html<br>2. Anti-PPARα antibody https://www.abcam.cn/products/primary-antibodies/ppar-alpha-antibody-ab215270.html<br>3. Anti- CD36 antibody https://www.abcam.cn/products/primary-antibodies/cd36-antibody-epr6573-ab133625.html<br>4. FABP1 Polyclonal antibody https://www.ptgcn.com/products/FABP1-Antibody-13626-1-AP.htm<br>5. CPT1A antibody https://www.cellsignal.cn/products/primary-antibodies/cpt1a-d3b3-rabbit-mab/12252?site-search-type=Products&N=4294956287&Ntt=%2312252s&fromPage=plp&_requestid=156528<br>6. CPT2 Polyclonal antibody https://www.ptgcn.com/products/CPT2-Antibody-26555-1-AP.htm<br>7. HMGCS2 antibody https://www.cellsignal.cn/products/primary-antibodies/hmgcs2-d3u1a-rabbit-mab/20940?site-search-type=Products&N=4294956287&Ntt=%2320940s&fromPage=plp&_requestid=157911<br>8. NF-κB p65 antibody https://www.cellsignal.cn/products/primary-antibodies/nf-kb-p65-d14e12-xp-rabbit-mab/8242?site-search-type=Products&N=4294956287&Ntt=%238242s&fromPage=plp&_requestid=158147<br>9. Phospho-NF-κB p65 antibody https://www.cellsignal.cn/products/primary-antibodies/phospho-nf-kb-p65-ser536-93h1-rabbit-mab/3033?site-search-type=Products&N=4294956287&Ntt=%233033s&fromPage=plp&_requestid=158281<br>10. CRM1 antibody https://www.scbt.com/p/crm1-antibody-c-1?requestFrom=search<br>11. RAN antibody https://www.scbt.com/p/ran-antibody-a-7?requestFrom=search<br>12. GAPDH Monoclonal antibody https://www.ptgcn.com/products/GAPDH-Antibody-60004-1-Ig.htm<br>13. Anti- Lamin B1 antibody https://www.abcam.cn/products/primary-antibodies/lamin-b1-antibody-epr22165-121-ab229025.html<br>14. β-Actin antibody https://www.cellsignal.cn/products/primary-antibodies/b-actin-13e5-rabbit-mab/4970?site-search-type=Products&N=4294956287&Ntt=%234970&fromPage=plp<br>15. Mouse monoclonal anti-Flag https://www.sigmaaldrich.cn/CN/zh/search/f1804?focus=products&page=1&perpage=30&sort=relevance&term=f1804&type=product<br>16. HA tag Polyclonal antibody https://www.ptgcn.com/products/HA-tag-Antibody-51064-2-AP.htm<br>17. Rabbit Control IgG https://abclonal.com.cn/catalog/AC005<br>18. Mouse Control IgG https://abclonal.com.cn/catalog/AC011<br>19. HRP Goat Anti-Rabbit IgG (H+L) https://abclonal.com.cn/catalog/AS014<br>20. Anti-mouse IgG, HRP-linked Antibody https://www.cellsignal.cn/products/secondary-antibodies/anti-mouse-igg-hrp-linked-antibody/7076?site-search-type=Products&N=4294956287&Ntt=7076&fromPage=plp&_requestid=174702<br>21. ABflo® 594-conjugated Goat Anti-Rabbit IgG (H+L) https://abclonal.com.cn/catalog/AS039<br>22. ABflo® 488-conjugated Goat Anti-Mouse IgG (H+L) https://abclonal.com.cn/catalog/AS053<br>23. CRM1/XPO1 Rabbit mAb https://abclonal.com.cn/catalog/A19625<br>24. PPARA Polyclonal antibody https://www.ptgcn.com/products/PPARA-Antibody-15540-1-AP.htm |

## Eukaryotic cell lines

Policy information about cell lines and Sex and Gender in Research

| | |
|---|---|
| Cell line source(s) | AML12 cell line (SCSP-550) was purchased from National Collection of Authenticated Cell Cultures (Shanghai, China). RAW264.7 (TIB-71) and HEK293T (CRL3216) cell lines were obtained from ATCC. |
| Authentication | AML12, RAW264.7 and HEK293T cell lines were authenticated by STR analysis and cell morphology. |
| Mycoplasma contamination | All the cell lines were tested negative for mycoplasma contamination. |
| Commonly misidentified lines (See ICLAC register) | No commonly misidentified cell lines were used in this study. |

## Animals and other research organisms

Policy information about studies involving animals; ARRIVE guidelines recommended for reporting animal research, and Sex and Gender in Research

| | |
|---|---|
| Laboratory animals | 5-week-old C57BL/6J male mice, 8-weeks-old C57BL/6N male mice ,5-week-old C57BL/6J male ob/ob mcie, and 8-week-old Pparα-/- male mice (C57BL/6N background), 8-week-old Pparαflox/flox male mice (C57BL/6J background) were used in this study. |
| Wild animals | No wild animals were used in this study. |
| Reporting on sex | Sex was not considered in this animal study design and analysis. Because NAFLD is male predominant and diet-induced fatty liver in female mice is very mild, only male mice are used in this study |
| Field-collected samples | No samples collected from the field. |

| | |
|---|---|
| Ethics oversight | All experiments were conducted with approval from Institutional Animal Ethics Committee of Shanghai University of Traditional Chinese Medicine. |

Note that full information on the approval of the study protocol must also be provided in the manuscript.
